# An Experimental Investigation on the Mechanical Performance of Engineered Cementitious Composites with Different Types of Steel Fibers

**DOI:** 10.3390/ma18132990

**Published:** 2025-06-24

**Authors:** Mohammad Maldar, Reza Kianoush, Hocine Siad, Mohamed Lachemi

**Affiliations:** Department of Civil Engineering, Toronto Metropolitan University, Toronto, ON M5B 2K3, Canada; mmaldar@torontomu.ca (M.M.);

**Keywords:** engineered cementitious composites (ECCs), steel fibers, strain hardening, polyvinyl alcohol (PVA), toughness, ductility

## Abstract

Engineered cementitious composites (ECCs), known for their superior ductility and strain-hardening behavior compared to conventional concrete, have been predominantly studied with polyvinyl alcohol (PVA) fibers. However, the potential economic and technical advantages of incorporating steel fibers into ECCs have been largely overlooked in the literature. This study investigates the mechanical performance of ECC reinforced with different types of steel fibers, including straight, twisted, hooked, and hybrid fibers of different lengths, as compared to PVA. The inclusion of various supplementary cementitious materials (SCMs) such as slag and fly ash with each type of steel fiber was also considered at a constant fiber volume fraction of 2%. The mechanical properties were assessed through compressive strength, splitting tensile strength, and four-point flexural tests along with calculations of toughness, ductility, and energy absorption capacity indices. This study compares the mechanical properties of different ECC compositions, revealing that ECCs with hybrid steel fibers (short and long) achieved more than twice the tensile strength, 12.7% higher toughness, and 36.4% greater energy absorption capacity compared to ECCs with PVA fibers, while exhibiting similar multiple micro-cracking behavior at failure. The findings highlight the importance of fiber type and distribution in enhancing an ECC’s mechanical properties, providing valuable insights for developing more cost-effective and resilient construction.

## 1. Introduction

Concrete, renowned for its widespread use in construction due to its cost effectiveness and ease of fabrication, is naturally brittle and prone to cracking, which can compromise its structural integrity. In response, there has been a significant interest towards rein-forcing concrete with fibers. The fiber-reinforced concrete (FRC) family, in summary, bifurcates into ultra-high-performance concrete (UHPC) [1] and engineered cementitious composites (ECCs) [2]. UHPC, reinforced with steel fibers, offers high compressive strength but typically achieves a tensile strain capacity not exceeding 0.5%, demonstrating strain-softening post-crack [3]. Conversely, ECCs, also known as strain-hardening cement-based composites (SHCC), are distinguished by their exceptional ductility and crack control capabilities. Garnering increased attention in research over the past decade, ECCs have emerged in response to the demand for more resilient and durable construction materials, developed through precise micromechanical modeling of the interaction between fibers and the cement matrix [4,5,6,7]. Unlike traditional composites, ECCs omit coarse aggregates to enhance their ductility, relying instead on a carefully calculated mix of discontinuous fibers. This composition, alongside meticulous selection of materials and processing techniques, enables ECCs to exhibit remarkable ductility. The evolution of ECCs from fiber-reinforced concrete (FRC) involves the strategic combination of fibers and matrix properties to ensure the composite transitions from brittle to ductile failure modes. Therefore, the choice of fibers and the composition of the binding material play pivotal roles in determining the mechanical behavior of an ECC, emphasizing a shift towards ductility in its failure response [8,9]. The unique characteristic of an ECC, setting it apart from conventional concrete and FRC, lies in its remarkable strain-hardening capacity ranging from 3 to 5%. This refers to an ECC’s strong ability to endure significant deformation even after cracking while still carrying increasing loads without a loss in strength. Under flexural testing, instead of failing abruptly like conventional concrete, the ECC specimen develops numerous fine cracks and continues to deform under stress. This unique performance in maintaining load-bearing capacity after crack formation is largely enabled by the development of multiple microcracks despite having a fiber volume fraction below 2% [10,11]. Contrarily, in FRC, the stress-bearing capacity diminishes following fiber rupture, which leads to crack propagation rather than the material’s ability to undergo strain hardening and distribute loads effectively, like with an ECC. The development and application of ECCs are poised to revolutionize the construction industry by offering materials that significantly extend the lifespan of infrastructure while mitigating the environmental impact through enhanced material efficiency.

The most commonly used fibers in ECCs are high-performance synthetic fibers such as polyvinyl alcohol (PVA) and polyethylene (PE) [12]. The cost of these synthetic fibers contributes to ECCs’ production expenses being significantly higher than those of normal concrete, with PVA accounting for 80% of the total cost of ECCs [13,14]. On the other hand, not every engineering project requires the advanced tensile strain capacity (over 3%) offered by ECCs. As a result, some researchers are exploring ways to optimize the balance between the fiber content and the mechanical properties of ECCs to make these more cost-effective for a broader range of applications. Abd Elmoaty et al. [15] investigated the use of polypropylene, polyolefin, steel (60 mm corrugated), and glass fibers as an economical and more readily available alternative to PVA fibers in ECCs. The authors particularly explored the effect of different fiber types and content on the mechanical behavior of different ECC compositions, although the study did not investigate the strain-hardening capabilities. Findings indicated that the application of these fibers, coupled with sustainable supplement materials like fly ash and furnace slag, could provide measures to produce ECCs with improved physical and mechanical characteristics, maintaining the goal of low costs and energy balance. Pan et al. [16] explored the feasibility of using unoiled and hybrid PVA fibers in ECC to reduce costs while maintaining tensile ductility and toughness. Through an extensive experimental program involving 21 mix proportions and tests like four-point bending, uniaxial tensile, and compressive tests, the study proposed three typical mixes of PVA-ECC that balance cost and performance. These mixes range from low-cost, lower tensile ductility reinforced by unoiled PVA fibers, to high-cost, high tensile ductility using oiled PVA fibers, aiming to make ECCs more affordable for widespread use in sustainable infrastructure development. Wang et al. [17] examined the influence of different volumes of polyethylene (PE) fibers on the mechanical and physical properties of ECC. They showed how varying PE fiber contents with a length of 18 mm (0%, 1%, 1.5%, and 2%) affects the composites’ ductility, strength, and strain-hardening behavior. The study revealed that a fiber volume fraction of 1% is critical for achieving desirable strain-hardening and high ductility in ECC, with a notable tensile strain capacity observed at higher fiber contents. In addition, a specimen with 2% of straight 8 mm steel fiber was tested. It showed a strain softening behavior post-crack similar to that of FRC. Sasmal and Avinash [18] explored the enhancement in mechanical properties in cementitious composites through the incorporation of PVA fibers. The study examined the effects of varying water–cement ratios, fiber volume fractions (1% and 2%), and fiber lengths (8 and 12 mm) on flexural strength and fracture behavior. The authors demonstrated that higher water–cement ratios facilitate chemical bonding and strain hardening, whereas lower ratios limit ductility despite providing high strength. Flexural strength increases with the increase in fiber volume fraction, but the increase in fiber length from 8 mm to 12 mm showed unfavorable results. This might be due to the lower water–cement ratio of 0.3, which was used to carry out the study with 12 mm PVA fibers. Said et al. [19] investigated the impact of PVA fibers on the toughness, compressive, and flexural strength of engineered cementitious composite (ECC) cubes and slabs, focusing on the reinforcing index as the key parameter. Through tests in direct tension and flexural toughness assessments and post-cracking strength techniques, the study found that compressive strength decreases with increasing reinforcing index. ECC PVA slabs exhibit strain-hardening and multiple cracking behaviors at higher reinforcing indices, although they did not achieve the desired ductility due to PVA fiber rupture.

According to the literature, the characteristics of steel fibers used in conventional FRC concretes can significantly impact their mechanical properties. For instance, Holschemacher et al. [20] presented an experimental study on the impact of various steel fiber types (50 mm hooked end and corrugated fibers) and amounts on the flexural and tensile strength, fracture behavior, and workability of high-strength concrete beams reinforced with steel bars. Utilizing different configurations of steel fibers and bar reinforcements, the research demonstrates that the type and geometry of fibers have a direct influence on ductility and load capacities post-cracking. However, due to the presence of coarse aggregate, the specimens showed strain softening post-crack. Kim et al. [21] investigated the flexural performance of Hybrid Ultra-High-Performance Fiber-Reinforced Concretes (UHPFRCs) utilizing different types of macro fibers, including long straight (30 mm), two types of hooked (30 and 62 mm), and twisted (30 mm) steel fibers, alongside a constant microfiber (straight 13 mm) type. It examined the enhancement in modulus of rupture, deflection capacity, and energy absorption capabilities across various fiber compositions, demonstrating that the flexural performance varies with the type of macro fiber used. The findings suggested that the blend of macro and microfibers significantly influences UHPFRC’s structural properties.

There is very limited research available on ECCs containing steel fibers, with no studies found on the effect of different types of steel fibers on the mechanical properties of ECCs. This lack of research presents a clear scientific gap, also highlighting a limited insight into how different matrix compositions, particularly the use of different supplementary cementitious materials (SCMs), interact with steel fibers to influence ECC performance. To address this significant research gap, this study provides valuable experimental data on the impact of various steel fibers including straight, twisted, short hooked, long hooked, and hybrid steel fibers, on ECC’s mechanical properties. The strain-hardening capabilities, failure modes, toughness, and flexural strengths of steel fibers-based ECCs were comparatively analyzed, and the effect of two SCMs, including granulated blast furnace slag (SL) and fly ash class-F (FF), was also considered. This comprehensive approach is intended to enhance the understanding of material behavior and potential improvements in ECC applications.

## 2. Experimental Program

### 2.1. Materials and Mixture Proportions

**Figure 1:**
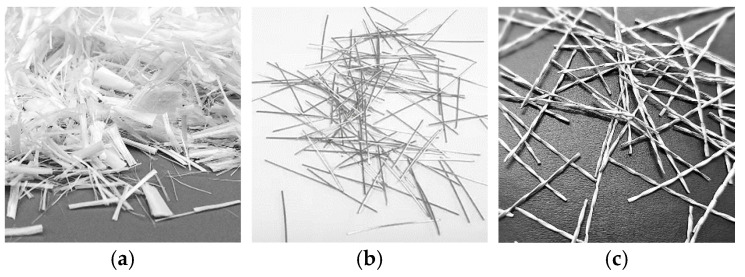

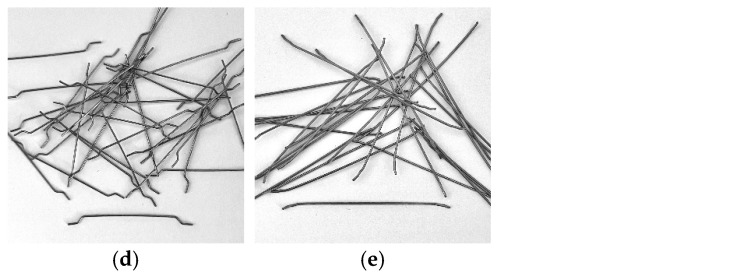
Fiber types and geometry, (**a**) polyvinyl alcohol fiber, (**b**) straight fiber, (**c**) twisted fiber, (**d**) short hooked fiber, (**e**) long hooked fiber.

ECC compositions differ by the Supplementary Cementitious Material (SCM) and fibers. Materials used for ECCs include Portland cement (PC) conforming to ASTM C150 Type I cement [22], ground granulated blast furnace slag (SL), class-F fly ash (FF), silica sand (SS) with average and maximum aggregate sizes of 150 μm and 400 μm, respectively, and high-range water-reducing admixture (HRWRA). The physical and chemical properties of PC, SL, and FF are shown in Table 1. Different fibers were used, including PVA and steel fibers in various shape and lengths, including 13 mm straight, 25 mm twisted, 30 mm hooked, and 60 mm hooked, along with hybrid 13 mm straight/30 mm hooked and 13 mm straight/60 mm hooked (Figure 1). The properties of these fibers are shown in Table 2. Nine different ECC compositions were cast, with the mix proportions shown in Table 3. All ratios are given as a percentage of cement weight, except for fiber, which is reported by fraction of total volume. The fiber volume fraction was kept at 2% in all mixtures. All ECCs were prepared with a constant FF/SL-to-PC ratio of 1.2, an SS-to-PC ratio of 0.8, and a water-to-cement ratio of 0.58. An appropriate amount of HRWRA was added to the mixture by targeting a minimum flow diameter of 18 mm after adding the fibers [23,24,25]. The amount of HRWRA mainly depended on the type of SCM in the mixture; approximate amounts of 0.0022 and 0.0052 gr/m^3^ were used for mixtures incorporated with FF and SL, respectively. In addition to ECCs, one batch of normal concrete (NC) was cast for better comparison. NC was prepared with 3.12, 3.45, and 0.5 for sand-, gravel-, and water-to-cement ratios, respectively. Fine and coarse aggregates used in NC had maximum sizes of 5 mm and 16 mm, respectively. A shear mixer was used to cast all ECC mixtures. After one day of casting, the specimens were left to cure in plastic bags for six days at 95% RH and 23 °C. Subsequently, they were moved to a laboratory medium for an additional 21 days at 50% RH and 23 °C.

### 2.2. Mechanical Testing

**Figure 2:**
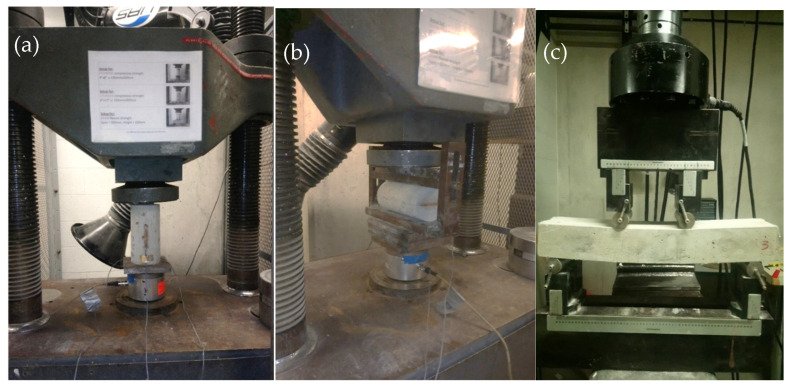
Apparatus for concrete property test, (**a**) compressive cylinder test, (**b**) cylinder splitting test, (**c**) four-point flexural test.

**Figure 3:**
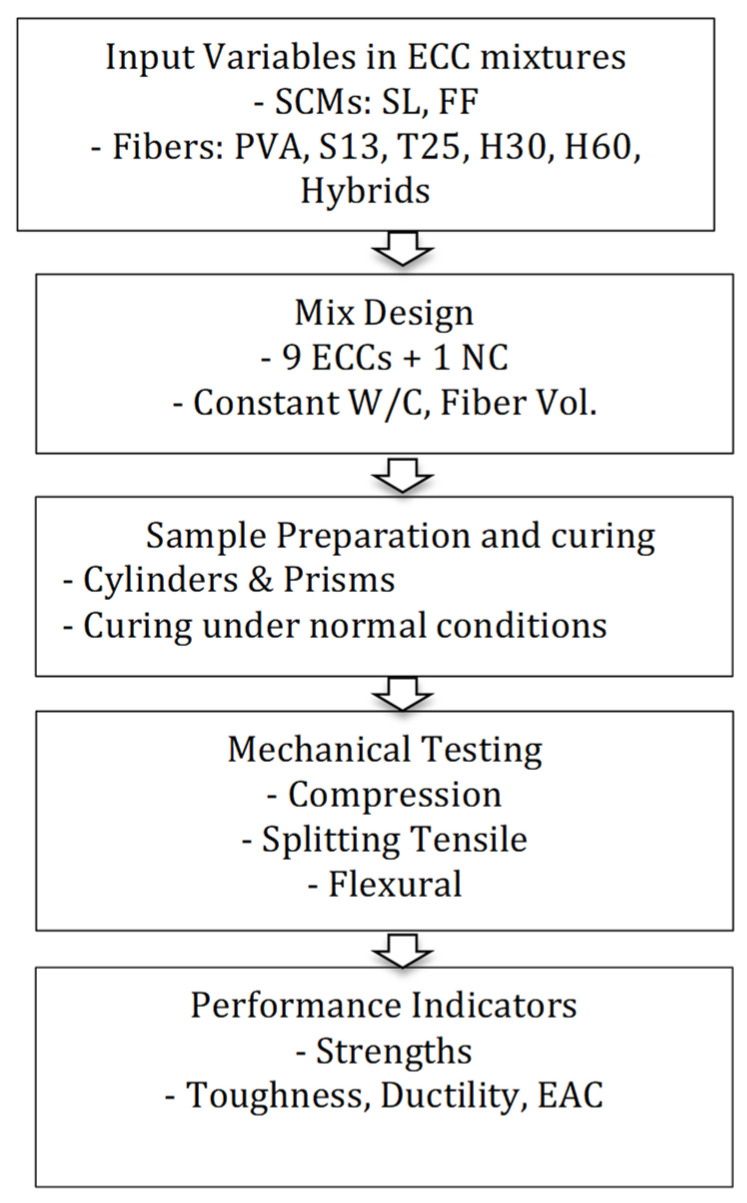
Flowchart summarizing the experimental program.

Three different material property tests were conducted on the samples, namely, the cylinder compressive test, the cylinder splitting test, and the four-point flexural test. These tests would give the compressive strength, the splitting tensile strength, flexural strength, ultimate mid-span deflection, toughness, ductility, and energy absorption capacity of ECCs. The apparatuses used for these tests are shown in Figure 2. The measurements were conducted in accordance with the ASTM standards [26,27,28] at the ages of 7 days and 28 days, with three specimens for each test. Prisms of 360 × 75 × 50 mm were cast for the four-point bending test to measure flexural strength and the corresponding mid-span deflection, toughness, ductility, and energy absorption capacity. Cylinders with a diameter of 100 mm and height of 200 mm were cast for the compressive and splitting tests. A flowchart presenting the sequence of the experimental program is shown in Figure 3.

## 3. Results

### 3.1. Compressive Strength

**Figure 4:**
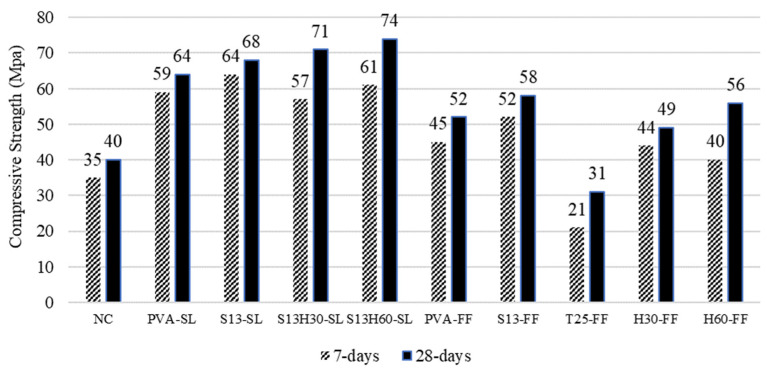
Compressive strength results of ECC mixtures.

Figure 4 shows the results of the compression tests at 7 and 28 days for all ten compositions. When considering the type of SCM in ECC, as can be seen from Figure 3, specimens incorporating slag showed higher compressive strength values than fly-ash-incorporated specimens. The behavior of slag-incorporated specimens would be explained by the chemical and physical properties of the SCMs. The amount of CaO, as the main ingredient for enhanced cementing behavior, is relatively high in the slag along with the substantially significant amount of SiO_2_, which is mostly responsible for the pozzolanic reactions at later ages. This implies that C-S-H gel formation could be expected to be accelerated in ECCs incorporating slag, which would significantly contribute to higher compressive strength. The other contributing factor for higher early-age compressive strength in ECC-SL would be the greater filling effect of SL particles (6.3 µm) that may have resulted in a denser microstructure than ECC-FF [29,30]. Also, the nucleation process, which depends predominantly on the active surface area of cementitious materials, may have been promoted with the use of SL, leading to accelerated hydration and enhanced mechanical strengths [31,32,33]. In the same FF-ECCs, by replacing PVA with S13 fiber, the compressive strength increases about 9%. However, when the fiber in the S13-FF composition is replaced by T25, H30, and H60, the compressive strength decreases by 46, 15, and 3%, respectively. This could be mainly due to the quality of fiber distribution in the specimens, since T25, H30, and H60 fibers are longer and have larger diameters compared to S13. This would result in poor fiber distribution in the matrix, which hinders the composition’s benefitting from fibers that have better bonding with concrete due to their shape. With the same fiber volume fraction, shorter fibers disperse more uniformly compared to longer fibers as shown by Yoo et al. [34] and Prathipati et al. [35]. Among ECCs with long steel fiber, T25-FF had the lowest compressive strength compared to H30-FF and H60-FF. This is likely due the lower tensile strength of T25 fiber and weaker bond with concrete due to its twisted shape compared to hooked H30 and H60 fibers [36]. On the other hand, when half of the fiber in S13-SL composition is replaced with H30 and H60, the compressive strength increases by 5 and 9%, respectively. Having a hybrid composition with microfibers (S13) and macrofibers (H30 or H60) likely resulted in a better fiber distribution, allowing the composition to fully benefit from the enhanced properties of H30 and H60 fibers. Hybrid compositions demonstrated highest compressive strength among all ECC compositions.

### 3.2. Splitting Tensile Strength

**Figure 5:**
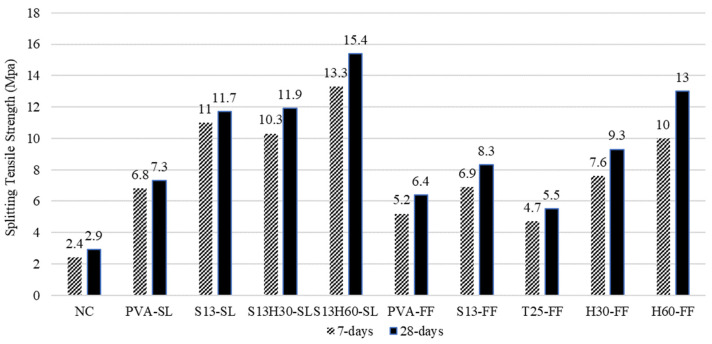
Splitting tensile strength results of ECC compositions.

The splitting tensile strength test was performed on 100 mm by 200 mm cylinders. The tensile strength of the compositions at 7 and 28 days is shown in Figure 5. The ECC specimens failed with less than 0.1 mm crack width, which indicates the crack tightness property of ECC. By replacing PVA with S13 fiber, tensile strength increased by 60 and 30% for SL- and FF-incorporated specimens, respectively. This is mainly due to the higher tensile strength of S13 fibers compared to PVA. Among ECCs with long steel fiber, T25-FF has the lowest tensile strength of 5.5 MPa compared to H30-FF and H60-FF with 9.3 and 13 MPa, respectively. This could be justified by the weaker bonding of T25 fiber with concrete compared to H30 and H60 [36]. The S13H60-SL hybrid composition achieved the highest tensile strength, 15.4 MPa, which is 2.1 times higher than PVA-SL. The balanced fiber volume fraction of short and long steel fibers resulted in better fiber distribution, allowing the composition to benefit from enhanced H30 and H60 fibers.

## 4. Flexural Behavior

### 4.1. Load–Deflection Response

**Figure 6:**
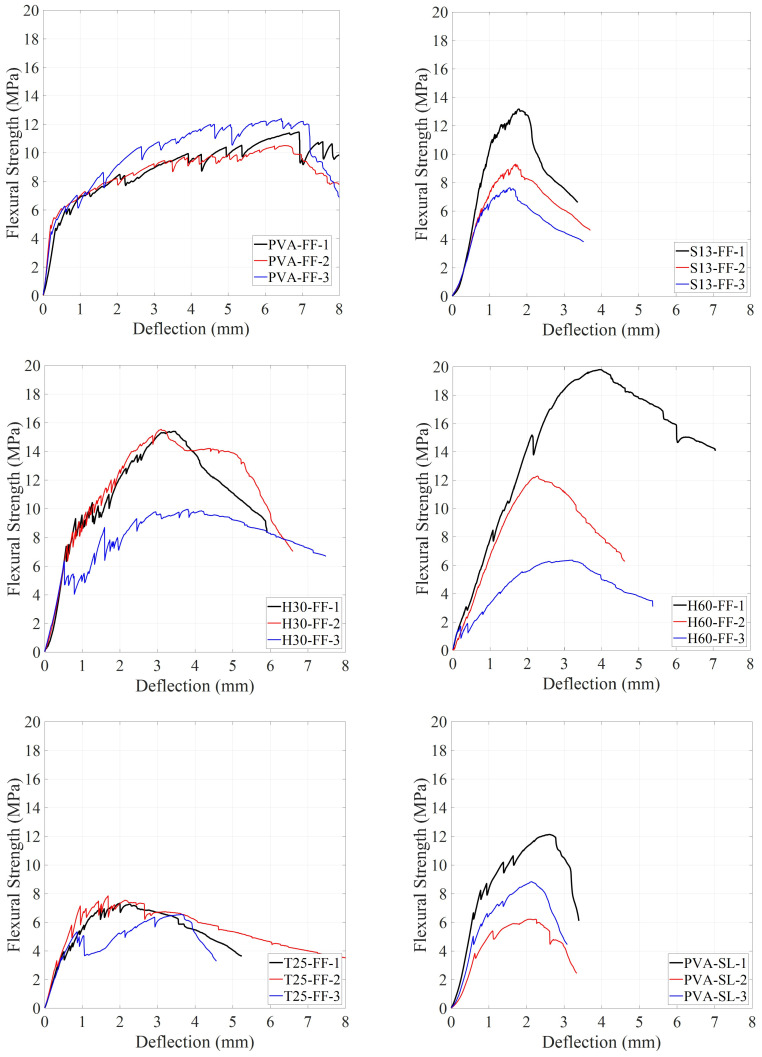

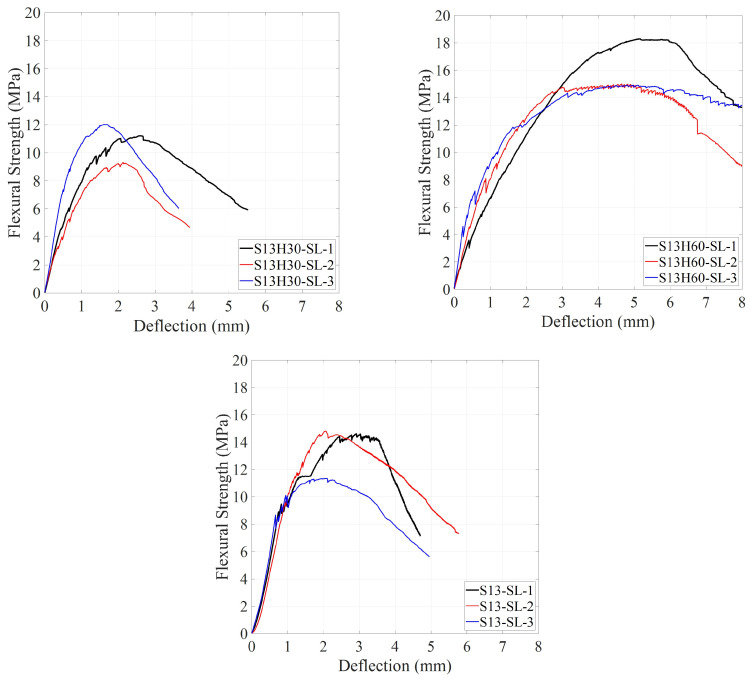
Flexural strength vs. mid-span defection of each ECC composition at 28 days.

Figure 6 shows the flexural strength versus the mid-span deflection of ECC specimens at 28 days. Different fibers and SCMs have notably impacted the flexural response of ECCs. Initially, before cracking occurred, the response curves were linear and directly proportional to the applied load, regardless of the fiber type or SCM. However, once the first crack appeared, the response became nonlinear. At this stage, the ECC matrix carried the increasing load, and the fibers resisted the pull-out effect after the initial cracking. At the cracked section, the entire load was carried by bridging fibers. With progressive loading, the additional tensile stress was transferred from the fibers to the composite through bond stresses. If these bond stresses do not exceed the ultimate bond strength, then additional cracks will be developed along the ECC prism [4]. All ECC compositions experienced strain-hardening behavior after the first crack regardless of the fiber type.

**Figure 7:**
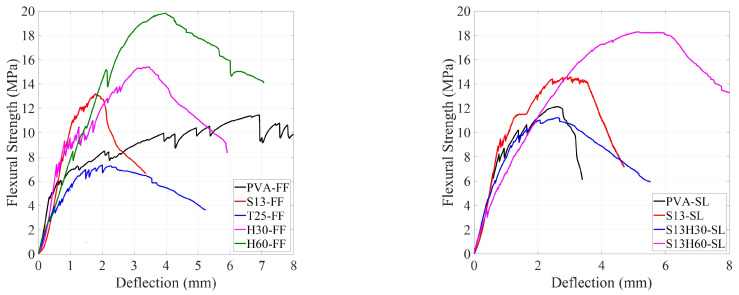
Flexural strength vs. mid-span defection for the best response of each ECC composition.

Figure 7 shows flexural strength versus mid-span deflection for the best response of each ECC composition. Peak flexural strength (f_MOR_), with its corresponding deflection (δ_MOR_), and first-crack flexural strength (f_LOP_), with its corresponding deflection (δ_LOP_), are recorded in Table 4. ECCs with PVA fibers showed a peak flexural strength (f_MOR_) of about 12 MPa, with δ_MOR_ values of 2.6 and 6.9 mm for PVA-SL and PVA-FF, respectively. While the δ_MOR_ of PVA-FF was significantly larger than that of PVA-SL, only PVA-FF demonstrated an extreme ductility with multiple micro-cracking behavior. In addition, both PVA-FF and PVA-SL showed multiple micro-cracking behavior at 7 days, whereas only PVA-FF showed multiple micro-cracking at 28 days. This could be attributed to the difference in the rate of advanced pozzolanic reactions between slag and fly ash. As the rate of pozzolanic reaction is higher in slag-incorporated specimens, a stronger bond will form between PVA fiber and the matrix [37,38]. This will result in fiber rupture rather than the fibers experiencing pull-out behavior and thus less micro-cracking behavior [39]. ECCs with S13 fiber showed an f_MOR_ of about 14 MPa, which is a 17% increase compared to ECCs with PVA. However, S13-FF and S13-SL recorded δ_MOR_ values of 1.8 and 2.9 mm, respectively, which are noticeably lower than the δ_MOR_ values of PVA-FF. Although both ECCs with S13 fiber showed good strain hardening after first crack, they failed to achieve the expected ECC behavior, such as high ductility with micro-cracking failure mode. The low aspect ratio and straight shape of S13 fibers resulted in a weaker bond between the fiber and the matrix, resulting in lower fiber bridging strength, which prevented S13-FF and S13-SL from demonstrating the micro-cracking behavior. By substituting S13 fibers with T25, peak flexural strength (f_MOR_) decreases by 45% compared to S13-FF and reaches 7.3 MPa with δ_MOR_ of 2.0 mm. T25-FF has the weakest performance among all ECC compositions. While T25 fibers have better bonding with the matrix than straight S13 fibers, they have lower tensile strength, resulting in lower fiber bridging strength. Additionally, the longer length of T25 negatively impacted the quality of fiber distribution in the specimens [40]. By substituting S13 fibers with H30, f_MOR_ increased by 17% compared to S13-FF, reaching 15.4 MPa with δ_MOR_ 3.5 mm. Higher tensile strength and better bonding with the matrix of hooked fibers compared to S13 and T25 clearly increased both f_MOR_ and δ_MOR,_ yet the ductile behavior of ECCs with PVA fibers still lacked. On the other hand, the H60-FF composition recorded an f_MOR_ of 19.8 MPa, a 50% increase compared to S13-FF, with δ_MOR_ of 4.0 mm, which is 2.2 times the δMOR of S13-FF. A good improvement can be seen in the H60-FF composition in terms of f_MOR_ and δ_MOR_. However, due to the longer length of fibers compared to H30, the H60-FF composition may have suffered from poor fiber distribution, causing the three samples of H60-FF to have different results under the flexural test. This improper fiber distribution of long fibers (60 mm) was already shown in the literature, particularly at a high volume fraction of 2% [35]. To overcome the issue of fiber distribution, ECCs with hybrid steel fibers were proposed. By having 1% volume fraction of S13 fibers with 1% volume fraction of H30/H60 fibers, the composition can benefit from enhanced properties of H30/H60 fibers while keeping the total volume fraction to 2% and not having fiber distribution issues. H60-SL and H30-SL recorded f_MOR_ values of 18.3 and 11.2 MPa, respectively, with δ_MOR_ values of 5.9 and 2.6 mm, respectively. Among ECCs with steel fiber, only S13H60-SL showed multiple micro-cracking behavior. The good synergy between short straight S13 fiber and long hooked H60 fiber resulted in a highly ductile ECC with only steel fibers and improved flexural strength. It worth mentioning that noticeable variabilities have been shown between the samples of some ECC mixtures, especially those of PVA-SL, S13-FF, and H60-FF (Figure 5). This is likely attributed to the random distribution and orientation of fibers within the ECC matrix. Fiber orientation significantly affects load transfer and crack-bridging mechanisms, especially in fiber-reinforced composites where mechanical performance relies on uniform fiber dispersion. In the case of PVA fibers, their short length and high bond strength may lead to uneven performance depending on alignment [41]. For steel fibers, especially the longer or hooked types used in S13-FF and H60-FF mixtures, variations in orientation and possible segregation during casting or flow-induced alignment could contribute to the observed dispersion [42]. Despite following consistent mixing and casting procedures, slight inconsistencies in fiber placement are difficult to avoid and may influence the test results. From a structural design perspective, this emphasizes the importance of considering fiber orientation effects, and further research involving fiber distribution analysis is recommended to better understand and control this variability.

### 4.2. Failure Mode and Micro-Cracking Behavior

**Figure 8:**
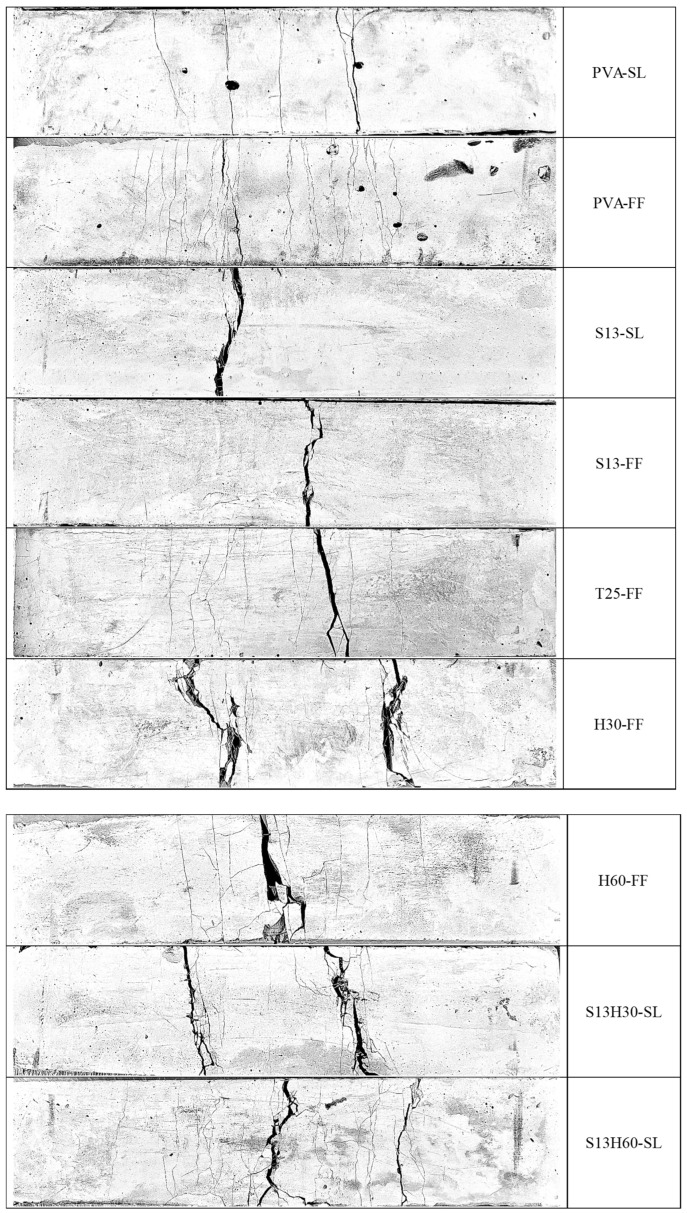
Bottom face of the failed ECC specimens under the four-point bending test at 28 days.

Under the flexural test, the ECC samples demonstrated ductile failures by developing cracks that propagated from the bottom of the prism throughout the depth. Figure 8 shows the cracking pattern at the bottom face of the failed ECC prisms at 28 days. Among ECCs with PVA, only PVA-FF showed multiple micro-cracking with a high δ_MOR_, as discussed in the previous section. ECCs with straight S13 fibers failed, showing a single wide crack without any micro-cracking. This indicates that as loading increases after the first crack, stresses do not redistribute to their neighboring sections, which would have resulted in micro-cracking, but rather redistribute through the depth of the section, which widens the single major crack. This is the result of the maximum fiber bridging capacity, which is associated with fiber rupture and/or pullout, being lower than the cracking strength of the matrix. For additional cracks to form in neighboring sections (micro-cracking), the fiber bridging capacity should remain higher than the cracking strength, which is known as the strength criterion [43]. ECCs associated with H30 fibers also showed minimum-to-no micro-cracking. This could be due to the high tensile strength of H30 fibers, which contributed to the cracking strength of the matrix. T25-FF and H60-FF showed limited micro-cracking behavior, having fiber distribution issues. Among all ECCs with steel fiber, only S13H60-SL showed multiple micro-cracking at failure, with high δ_MOR_ similar to that of the PVA-FF composition. The balanced fiber volume fraction of the combined S13 and H60 fibers resulted in good fiber distribution in the composition. Better bonding of H60 fibers with the matrix compared to S13 fibers along with a lower tensile strength than H30 fibers resulted in higher fiber bridging capacity than the cracking strength of the matrix for the S13H60-SL composition and subsequently micro-cracking behavior with high ductility.

### 4.3. Initial Stiffness and First-Crack Stress

**Figure 9:**
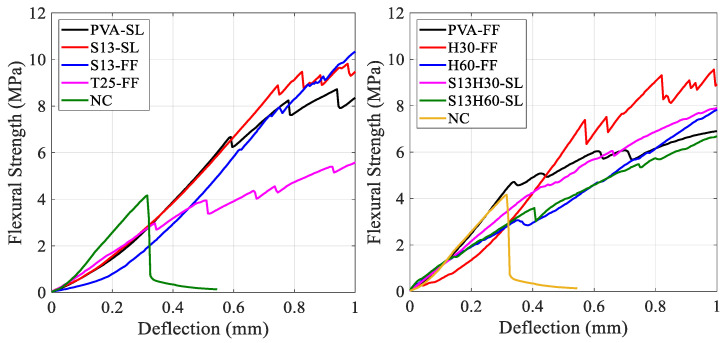
Flexural strength vs. mid-span defection of ECC compositions at 28 days.

Figure 9 shows the elastic portion of the flexural response of the different compositions. The initial stiffness (Ei) of compositions is determined by the slope of load–deflection curves and it is recorded in Table 4. In addition, the flexural limit of proportionality (f_LOP_) was identified as the point on the load–deflection curve where a deviation from linearity was first observed, indicating the end of the elastic behavior. In practice, this was determined by drawing a tangent to the initial linear portion of the curve and identifying the point at which the actual curve began to diverge from this tangent. For series such as S13H30-SL, where the transition is more gradual, the f_LOP_ value was obtained through a combination of visual inspection and analysis of the change in the slope to ensure consistency in interpretation across all series. The highest initial stiffness was achieved by H30-FF, PVA-FF, and NC, with Ei values of 11.3, 10.9, and 9.4 (kN/mm), respectively, while the lowest initial stiffness was achieved by H60-FF, S13H60-SL, and T25-FF, with Ei values of 4.9, 5.1, 5.6 (kN/mm), respectively. In terms of first-crack stress (f_LOP_), the highest first-crack stress was achieved by S13-SL, S13-FF, and H30-FF, with f_LOP_ values of 8.9, 8.0, and 7.3 (MPa), respectively, while the lowest first-crack stress was achieved by S13H60-SL, H60-FF, and T25-FF, with f_LOP_ values of 3.5, 3.1, and 3.0 (MPa), respectively. It is interesting to note that S13H60-SL and H60-FF have low cracking stress with low initial stiffness compared to other compositions. This is in line with the strength criterion and one of the reasons for the high ductility of these two compositions. On the other hand, S13-FF and S13-SL have the highest cracking stress, indicating high matrix cracking strength as opposed to the strength criterion. This can explain why in ECCs with S13 fibers, no micro-cracking is observed and the specimen failed under a single wide crack. In ECCs, after the first crack, the average specimen can carry a load of up to 2 to 8 times more than the flexural strength corresponding to the first crack [44,45].

### 4.4. Toughness, Ductility, and Energy Absorption Capacity

In order to better quantify the flexural behavior of ECC compositions, indices for toughness, ductility, and energy absorption capacity are evaluated in this section.

#### 4.4.1. Toughness Index

**Figure 10:**
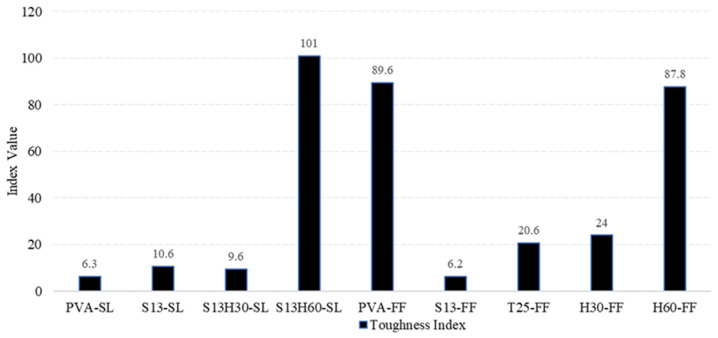
Toughness Tndex obtained for each ECC composition.

The Flexural Toughness Index (IT) evaluates the influence of fibers in the strain-hardening and post-crack behaviors of ECCs. Toughness shows the energy absorbed by the material and its ductility capacity, which can be calculated through the area under the load–deflection curve. There are different methods to express the Toughness Index up to a specified value of deflection. In this paper, Toughness Index (IT) is defined as the ratio of area under the load–deflection curve up to δ_MOR_ over the area under the load–deflection curve up to δ_LOP_ [46]. The related parameters need to be accurately determined based on load–deflection curve, which are recorded in Table 4. Figure 10 shows the obtained IT indices for each ECC composition. Notably, S13H60-SL (IT = 101.0), PVA-FF (89.6), and H60-FF (87.8) significantly outperform the other mixes, which fall below 24, with S13H60-SL presenting an IT more than 12.7% higher than that of the PVA-ECC. This disparity highlights two key factors: fiber geometry/type and matrix compatibility. Research shows that long, hooked-end steel fibers enhance energy absorption by providing mechanical anchorage and resisting pull-out during crack propagation [47]. The S13H60-SL mixture combines short and long steel fibers, creating a hybrid system that improves fiber distribution and crack bridging efficiency, resulting in the highest IT observed. Comparable benefits have been reported by Liu and Han (2024) [48], who linked hybrid fiber systems (steel + PVA) to improved bond behavior and energy dissipation in ECCs.

The matrix type also plays a critical role. The SL-based matrix appears to have higher cohesiveness and toughness than the FF mixture, in line with the compressive and tensile strength results. Furthermore, recent findings confirm that steel fibers consistently outperform PVA in toughness enhancement due to their higher strength and pull-out resistance. However, PVA fibers contribute to ductility through crack-bridging and finer crack control [49].

In addition, the low first-crack stress (f_LOP_) of H60-FF and S13H60-SL, along with their capacity to undergo large deformations without a significant loss of strength, may be additional factors contributing to their superior Toughness Index compared to the other compositions.

#### 4.4.2. Ductility Index

**Figure 11:**
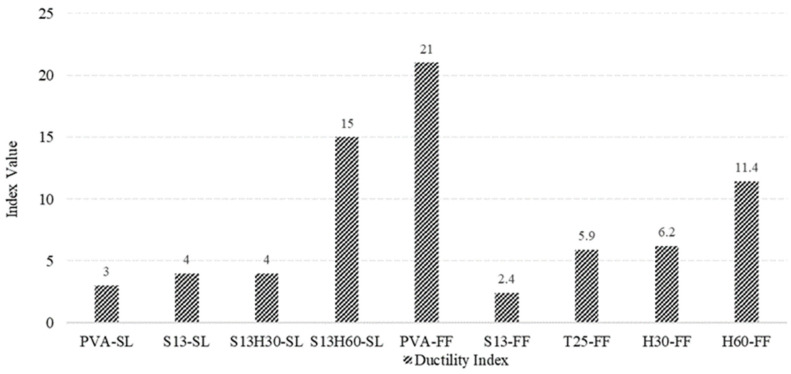
Ductility Index obtained for each ECC composition.

The Ductility Index (ID) can directly measure the ductility of the material and is defined as the ratio of δ_MOR_ to δ_LOP_ [50]. The Ductility Index indicates the ability of a material to undergo inelastic deformations without loss of strength. A ductile behavior is a crucial feature to prevent the brittle failure of members, ensuring large inelastic deformations. Figure 11 shows that the highest ID values were observed in PVA-FF (21.0), S13H60-SL (15.0), and H60-FF (11.4). Fiber type significantly influences ductility. PVA fibers like those in PVA-FF offer excellent bond to the matrix and help promote multiple fine cracks under load, resulting in strain hardening and superior ductility [51]. This mechanism explains why the PVA-FF composition exhibits the highest ID.

In steel-fiber-reinforced mixes, the hybrid S13H60-SL achieved the second-highest ID, which can be attributed to its mixture of short and long hooked fibers. This configuration offers both good dispersion and enhanced bridging capacity, similar to findings in hybrid PVA–steel ECC studies that show improved energy absorption and ductility [52]. Additionally, the SL matrix appears more cohesive, facilitating better stress transfer than the more porous FF matrix. Conversely, S13-FF recorded the lowest ID (2.4). This is likely due to the limitations of short, straight steel fibers, which have weaker mechanical anchorage and less effective bridging across cracks. The high cracking stress of S13-FF and low fiber bridging capacity of S13 fibers make the composition incapable of redistributing stress to neighboring sections (micro-cracking), thus experiencing large inelastic deformations. Similar observations have been reported by Venkateswarlu and Gunneswara (2023) [53] in alkali-activated concretes where short steel fibers yielded lower Ductility Indices.

#### 4.4.3. Energy Absorption Capacity

**Figure 12:**
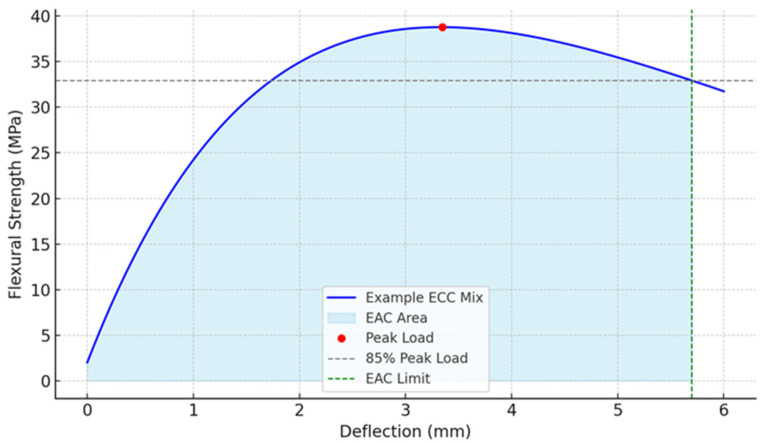
Method for calculating energy absorption.

**Figure 13:**
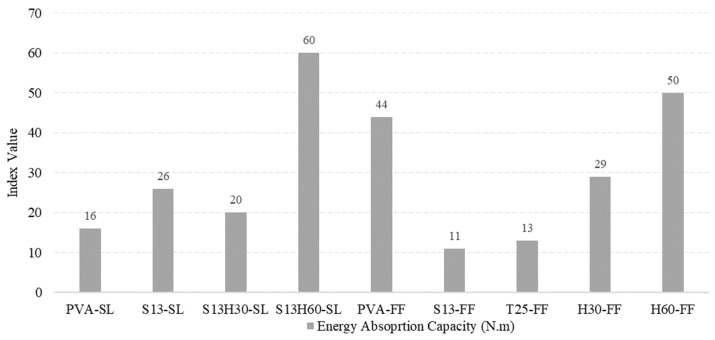
Energy absorption capacity indices obtained for each ECC composition.

The Energy Absorption Capacity (EAC) can directly calculate the energy absorbed by the material. The EAC is the area under the load–deflection curve up to a limiting deflection point [54]. In this study, the EAC is calculated as the area under the load–deflection curve from the origin up to the point where the load dropped to 85% of the peak load, after the maximum load has been reached (Figure 12). Figure 13 shows the EAC indices for each ECC composition. The highest energy absorption was observed for S13H60-SL (60 N·m), H60-FF (50 N·m), and PVA-FF (44 N·m), which also ranked highest in the Toughness and Ductility Indices, confirming their superior mechanical performance. Importantly, S13H60-SL presented an EAC around 36.4% higher than that of the control PVA-FF. These results clearly highlight the significant effect of both fiber type and matrix composition on energy absorption. Steel fibers, particularly long and hooked-end types, are known for their ability to resist pull-out and maintain bridging across wide cracks, which directly contributes to increased energy absorption capacity. The H60-FF and S13H60-SL mixes demonstrate this effect. The hybrid S13H60-SL mixture, which combines short and long hooked steel fibers, benefits from improved fiber dispersion and crack-bridging synergy, leading to the highest recorded EAC. Similar findings have been reported by Li et al. (2022), who showed that hybrid PVA–steel fiber systems enhanced energy dissipation through improved crack control and fiber anchorage [55]. In contrast, PVA fibers, despite having excellent crack-bridging capabilities, are limited in tensile strength compared to steel. While they enable strain-hardening and multi-cracking behaviors, they absorb less energy per unit of deformation. This explains why PVA-FF, although superior in ductility, slightly lags behind steel-based mixes in terms of EAC. As noted by Chen et al. (2024), while PVA fibers are excellent at controlling crack width and promoting ductile behavior, these generally yield slightly lower energy absorption compared to steel-based mixes under comparable conditions [56]. In addition, the SL matrix provided a more cohesive environment for higher effective stress transfer at the fiber–matrix interface. This is evident in the S13H60-SL mixture, where the hybrid fiber system performed optimally without significant fiber clustering. In contrast, the FF matrix, used in the H60-FF mix, posed challenges for fiber bonding with long steel fibers. The reduced workability may have also compromised consistency in performance, as previously observed by da Silva Neto et al. (2025) [57]. Although H60-FF shows desirable results, it is practically challenging to cast this composition as long fiber length and 2% fiber volume fraction resulted in fiber distribution issues, which is the same issue encountered with the T25 and H30 fibers. The fiber distribution issue was resolved in S13H60-SL since 1% fiber volume fraction of H60 was used, resulting in S13H60-SL outperforming PVA-FF in all criteria except for the Ductility Index.

## 5. Conclusions

ThIS comprehensive experimental investigation into the mechanical properties of engineered cementitious composites (ECCs) with different types of steel fibers has provided insights into evaluating ECC formulations for enhanced ductility, strength, and overall mechanical performance. The findings of this study can be summarized as follows:

- Hybrid compositions achieved the highest compressive and tensile strengths among all tested ECCs, surpassing traditional PVA-ECCs and other steel fiber ECCs.
- ECCs with S13 fibers failed with a single crack without micro-cracking behavior and poor ductility. The high cracking strength, along with A low aspect ratio and straight shape, of S13 fibers resulted in a weaker bond between the fiber and the matrix, resulting in lower fiber bridging strength, which prevented S13-FF and S13-SL from demonstrating the desirable micro-cracking behavior and ductility.
- Single-fiber ECCs with longer steel fibers faced challenges in fiber distribution, leading to reduced performance in compositions such as H30-FF, H60-FF, and T25-FF.
- S13H60-SL demonstrated exceptional toughness, ductility, and energy absorption capacity. The combination of short and long steel fibers resolved the fiber distribution issue and showed multiple micro-cracking behavior with the highest compressive and tensile strengths among all the samples.
- ECCs with PVA fibers exhibited exceptional ductility and multiple micro-cracking behavior, although only the hybrid S13H60-SL composition among the steel fiber ECCs achieved a similar ductility and micro-cracking behavior, making it the most effective steel fiber-reinforced ECC in this study.
- The findings suggest that carefully designed hybrid steel fiber ECCs can outperform traditional ECCs with PVA fibers, providing a more cost-effective and resilient material option for the construction industry.

It is also worth highlighting that this study primarily focused on a comprehensive evaluation of the mechanical properties of fiber-reinforced ECC mixtures incorporating various types of steel fibers compared to PVA fibers. The analysis and discussion were guided by the experimental findings. However, to facilitate the practical implementation and wider adoption of these materials, further research is recommended to compare ECC mixtures with conventional concrete incorporating the same binder composition and/or fiber types. Future studies should also investigate the structural behavior of ECCs with hybrid steel fibers at the element level, such as beams, slabs, and columns, to better assess their practical applicability in real-world construction. Further optimization of fiber combinations and volume fractions may also enhance both performance and workability.

## Figures and Tables

**Table 1:** Chemical and physical properties of OPC, slag and fly ash.

Chemical Composition (%)	PC	FF	SL
SiO_2_	19.5	57	36.8
Al_2_O_3_	5.1	21	8.72
Fe_2_O_3_	2.95	4.2	0.55
MnO	-	0.02	0.33
MgO	2.5	1.8	11
CaO	61.8	9.8	38.1
Na_2_O	0.3	2.2	0.21
K_2_O	1.11	1.5	0.31
TiO_2_	-	1.05	0.37
SiO_2_ + Al_2_O_3_ + Fe_2_O_3_	27.5	82.2	46
Loss on ignition (LOI)	2.5	1.74	1.1
Specific gravity (g/cm^3^)	3.1	2.25	3.1

**Table 2:** Mix proportions of the mixtures (units are in kg/m^3^).

Type of Fiber	Shape	Length (mm)	Diameter (mm)	Aspect Ratio	Tensile Strength (MPa)
PVA	straight	8	0.038	210	1600
S13	straight	13	0.2	65	1900
T25	twisted	25	0.25	100	1700
H30	hooked	30	0.38	80	3070
H60	hooked	60	0.75	80	2200

**Table 3:** Mix proportions of ECC mixtures.

Composition Notation	Fiber	Cement	Silica Sand	Water	Slag	Fly Ash	Fiber (Vol. Fraction)
PVA_SL	PVA	1	0.8	0.58	1.2	-	0.02
PVA_FF	PVA	1	0.8	0.58	-	1.2	0.02
S13_SL	13 mm straight	1	0.8	0.58	1.2	-	0.02
S13_FF	13 mm straight	1	0.8	0.58	-	1.2	0.02
T25_FF	25 mm Twisted	1	0.8	0.58	-	1.2	0.02
H30_FF	30 mm hooked	1	0.8	0.58	-	1.2	0.02
H60_FF	60 mm hooked	1	0.8	0.58	-	1.2	0.02
S13H30_SL	13 mm straight 30 mm hooked	1	0.8	0.58	1.2	-	0.02
S13H60_SL	13 mm straight 60 mm hooked	1	0.8	0.58	1.2	-	0.02

**Table 4:** Summary of flexural parameters for each ECC composition.

	*f*_LOP_ (MPa)	δ_LOP_ (mm)	*f*_MOR_ (MPa)	δ_MOR_ (mm)	E_i_ (kN/mm)
PVA-SL	6.7	0.85	12.4	2.6	9.1
PVA-FF	4.7	0.33	11.5	6.9	10.9
S13-SL	8.9	0.74	14.6	2.9	9.0
S13-FF	8.0	0.75	13.2	1.8	9.1
T25-FF	3.0	0.34	7.3	2.0	5.6
H30-FF	7.3	0.56	15.4	3.5	11.3
H60-FF	3.1	0.35	19.8	4.0	4.9
S13H30-SL	6.0	0.66	11.2	2.6	6.8
S13H60-SL	3.5	0.40	18.3	5.9	5.1

## Data Availability

The original contributions presented in this study are included in the article. Further inquiries can be directed to the corresponding author.
